# Hexagonal Boron Nitride for Photonic Device Applications: A Review

**DOI:** 10.3390/ma16052005

**Published:** 2023-02-28

**Authors:** Shinpei Ogawa, Shoichiro Fukushima, Masaaki Shimatani

**Affiliations:** Advanced Technology R&D Center, Mitsubishi Electric Corporation, 8-1-1 Tsukaguchi-Honmachi, Amagasaki 661-8661, Hyogo, Japan

**Keywords:** hexagonal boron nitride, hBN, two-dimensional materials, photonics

## Abstract

Hexagonal boron nitride (hBN) has emerged as a key two-dimensional material. Its importance is linked to that of graphene because it provides an ideal substrate for graphene with minimal lattice mismatch and maintains its high carrier mobility. Moreover, hBN has unique properties in the deep ultraviolet (DUV) and infrared (IR) wavelength bands owing to its indirect bandgap structure and hyperbolic phonon polaritons (HPPs). This review examines the physical properties and applications of hBN-based photonic devices that operate in these bands. A brief background on BN is provided, and the theoretical background of the intrinsic nature of the indirect bandgap structure and HPPs is discussed. Subsequently, the development of DUV-based light-emitting diodes and photodetectors based on hBN’s bandgap in the DUV wavelength band is reviewed. Thereafter, IR absorbers/emitters, hyperlenses, and surface-enhanced IR absorption microscopy applications using HPPs in the IR wavelength band are examined. Finally, future challenges related to hBN fabrication using chemical vapor deposition and techniques for transferring hBN to a substrate are discussed. Emerging techniques to control HPPs are also examined. This review is intended to assist researchers in both industry and academia in the design and development of unique hBN-based photonic devices operating in the DUV and IR wavelength regions.

## 1. Introduction

**Figure 1:**
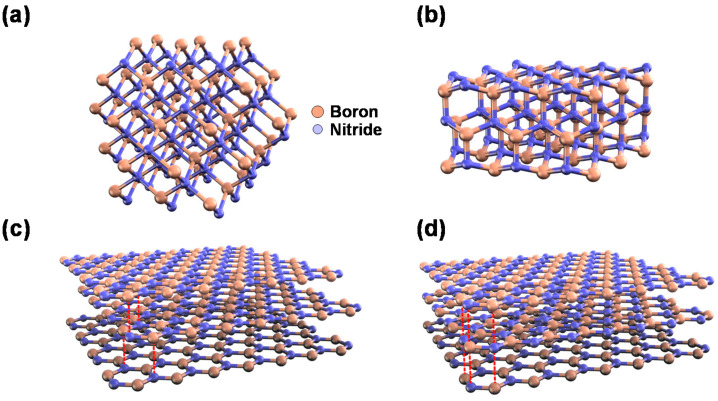
Schematic crystal structures of (**a**) cubic boron nitride (cBN), (**b**) wurtzite boron nitride (wBN), (**c**) rhombohedral boron nitride (rBN), and (**d**) hexagonal boron nitride (hBN).

Boron nitride (BN) is a III-nitride compound of boron and nitrogen [1]. It has four main crystalline forms, namely cubic BN (cBN; Figure 1a), which is analogous to diamond; wurtzite BN (wBN; Figure 1b), which is analogous to lonsdaleite (hexagonal diamond); rhombohedral BN (rBN; Figure 1c), which is analogous to graphite; and hexagonal BN (hBN; Figure 1d), which is also analogous to graphite, but with a different stacking structure as compared with rBN [2].

**Figure 2:**
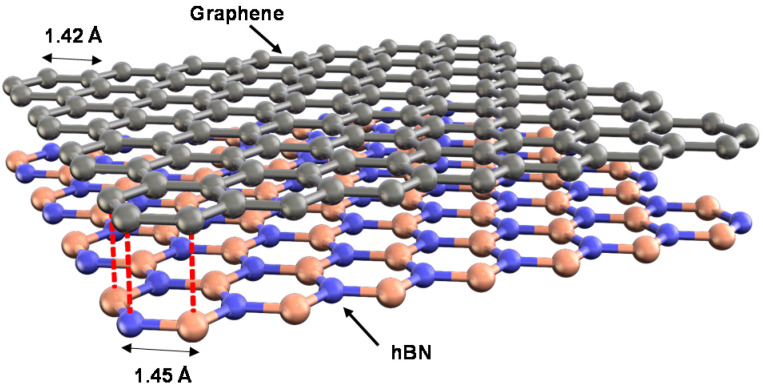
Lattice matching of graphene and hBN.

hBN is a wide-bandgap semiconductor with high thermal conductivity and chemical inertness and is expected to aid the development of novel devices for application under extreme conditions such as high temperature. Notably, hBN layers have a honeycomb lattice structure based on sp^2^ hybridization (Figure 2), which is similar to that of graphene.

The surface of hBN is atomically smooth and free of dangling bonds and charge-trapping sites. Therefore, it has attracted considerable interest as an ideal substrate for forming van der Waals (vdW) heterostructures with graphene and other two-dimensional (2D) materials. In contrast to conventional substrates with insulating layers such as SiO_2_, hBN substrates enable the full electronic and optical properties of 2D materials to be exploited. For example, the carrier mobility of graphene on hBN can reach that of suspended graphene [3], and hBN can support the highly confined low-loss surface plasmon polaritons (SPPs) of graphene [4].

In addition to its use as a substrate material for 2D vdW heterostructures, hBN has promising photonic properties owing to its unique bandgap structure and hyperbolic phonon polaritons (HPPs). For instance, various deep ultraviolet (DUV) devices have been designed based on the wide-bandgap structure of hBN, and infrared (IR) perfect absorbers, sub-diffraction imaging sensors, and chemical sensors have been designed based on its HPPs.

There are a number of excellent reviews on hBN; however, they typically focus either on its fundamental physics [5,6,7,8], synthesis [9,10], and vdW heterostructures [11,12]. Herein, we review hBN as a 2D material for application in photonic devices considering its unique bandgap structure and HPPs. The advantages of hBN-based photonic devices are described, and the future challenges are presented. The use of hBN in photon emitters is not covered here, because its mechanism and applications differ from those of the abovementioned photonic applications. The reader is referred to other important review papers in this regard [13,14]. The importance of hBN is rapidly growing in various photonic applications. This review paper aims to provide the fundamental physics of hBN devices and outlines of various photonic application fields to aid the development of novel photonic devices using hBN.

## 2. Theoretical Background of Optical Properties of hBN

### 2.1. Electronic Bandgap Structure

The bandgap structure of hBN has long been debated. Theoretical calculations, such as first-principles calculations, show that it should have an indirect bandgap structure [15,16,17,18], whereas experimental results using optical microscopy indicate that the obtained optical properties are caused by a direct bandgap [19,20,21]. For example, Watanabe et al. fabricated high-purity hBN crystals and observed an intense luminescence peak at 5.76 eV under UV irradiation, which they attributed to a direct bandgap [20]. This issue was ultimately resolved using two-photon excitation, which revealed that multilayer hBN has an indirect bandgap at 5.955 eV [22]. Its intense luminescence is attributed to strong exciton–phonon interactions that produce self-trapped exciton states owing to the vdW structure of hBN [23].

**Figure 3:**
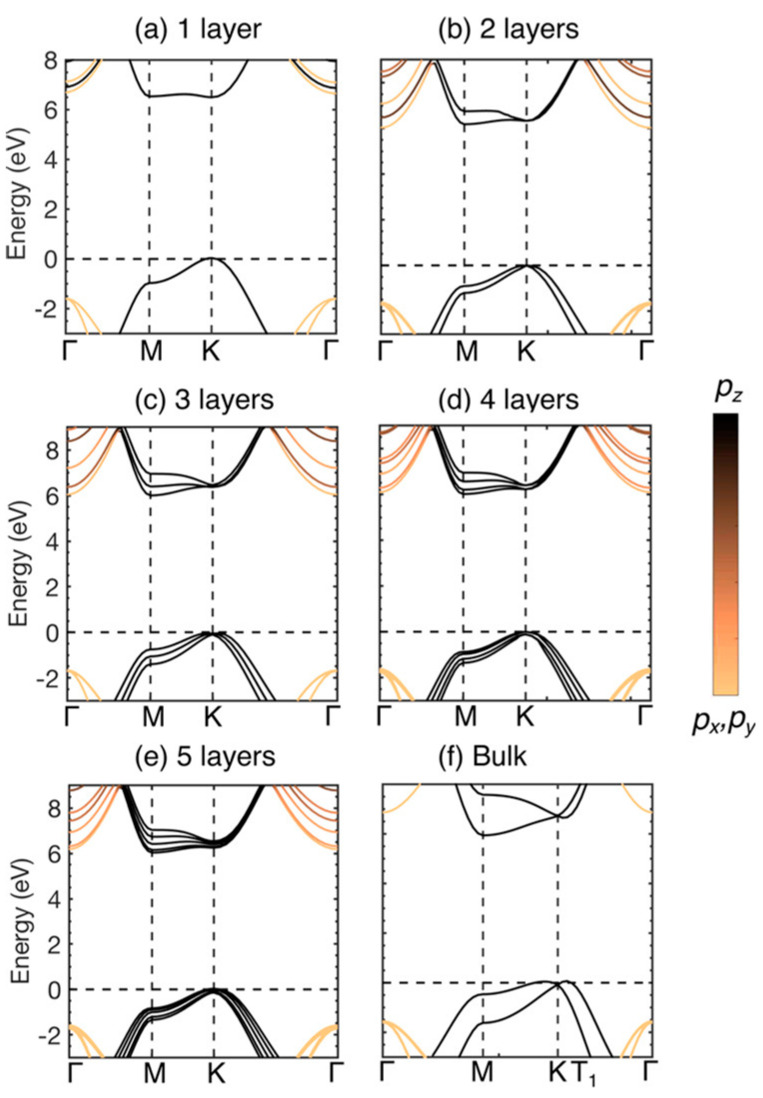
Evaluated bandgap structure of hBN with (**a**) one, (**b**) two, (**c**) three, (**d**) four, and (**e**) five layers, and (**f**) bulk hBN. Figures are adapted with permission from [24]. Copyright 2018 American Chemical Society.

As shown in Figure 3, the bandgap structure of hBN strongly depends on the layer number [24]. The bandgap structure was investigated by first-principles calculations. Monolayer hBN exhibits a direct bandgap; however, when the layer number is greater than or equal to two, hBN exhibits an indirect bandgap. This bandgap structure means that hBN can be applied in devices such as light-emitting diodes (LEDs) and photodetectors (PDs) operating in the DUV wavelength region. Furthermore, monolayer hBN is a promising platform for polariton manipulation in the UV range [25].

### 2.2. Asymmetric Permittivities

hBN has two anisotropic permittivities, *ε*_‖_ and *ε*_⊥_, which correspond to two types of phonon modes: (i) an out-of-plane (‖) mode with a transverse optical frequency (ω_TO_) of 780 cm^−1^ and longitudinal optical phonon frequency (ω_LO_) of 830 cm^−1^, and (ii) an in-plane (⊥) mode with a ω_TO_ of 1370 cm^−1^ and ω_LO_ of 1610 cm^−1^ [26]. Accordingly, the anisotropic permittivity of hBN is defined by [26]:

(1) $$\epsilon_{m}=\epsilon_{\infty ,\;m}+\epsilon_{\infty ,\;m}\times \frac{{\left(\omega_{\mathrm{LO},\;m}\right)}^{2}-{\left(\omega_{\mathrm{TO},\;m}\right)}^{2}}{{\left(\omega_{\mathrm{TO},\;m}\right)}^{2}-\omega^{2}-i\omega \Gamma_{m}},$$

where *ε*_∞_ and Γ are the high-frequency dielectric permittivity and damping constant, respectively; *m* = ‖ and ⊥; *ε*_∞,⊥_ = 4.87, *ε*_∞,‖_ = 2.95, Γ_⊥_ = 5 cm^−1^, and Γ_‖_ = 4 cm^−1^ [27]; and ω is the wave frequency.

**Figure 4:**
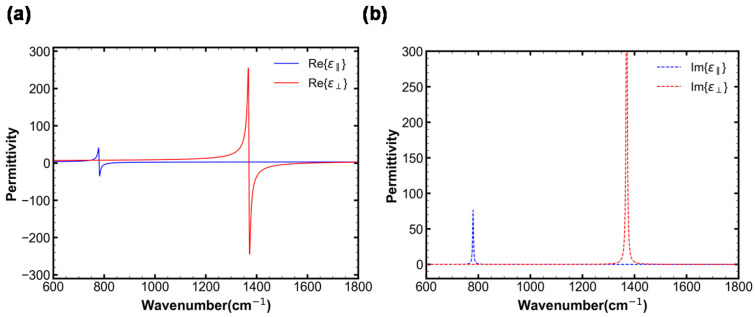
(**a**) Real and (**b**) imaginary parts of the permittivities of hBN.

The two anisotropic permittivities are the origin of the unique HPPs of hBN. Figure 4a,b show the real and imaginary parts of the permittivities of hBN, respectively. Two distinct types of reststrahlen (RS) bands are formed, in which the permittivities *ε*_‖_ and *ε*_⊥_ have opposite signs. Type-I (where *ε*_‖_ < 0 and *ε*_⊥_ > 0) occurs in the frequency range of 746–819 cm^−1^ and wavelength range of 12.21–13.4 μm, whereas Type-II (where *ε*_⊥_ < 0 and *ε*_‖_ > 0) occurs in the frequency range of 1370–1610 cm^−1^ and wavelength range of 6.21–7.30 μm. Therefore, hBN has HPPs in these two RS bands in the IR region at approximately 7 and 13 μm.

**Figure 5:**
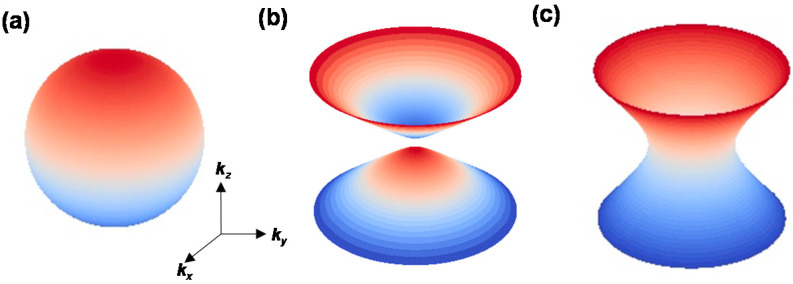
Isofrequency surfaces of (**a**) conventional (non-hyperbolic) materials (*ε*_‖_ = *ε*_⊥_ < 0), (**b**) Type-I hyperbolic materials (*ε*_‖_ < 0, *ε*_⊥_ > 0), and (**c**) Type-II hyperbolic materials (*ε*_⊥_ < 0, *ε*_‖_ > 0).

In these hyperbolic regions, the isofrequency surface with *z* as the optical axis is defined by [28]:

(2) $$\frac{k_{x}^{2}+k_{y}^{2}}{\epsilon_{\|}}+\frac{k_{z}^{2}}{\epsilon_{⊥}}={\left(\frac{\omega }{c}\right)}^{2},$$

where *k_x_*, *k_y_*, and *k_z_* are the wavevectors of the *x*, *y*, and *z* axes, respectively, and *c* is the speed of light in vacuum. Figure 5a–c show the isofrequency surfaces of non-hyperbolic materials (*ε*_‖_ = *ε*_⊥_ < 0), Type-I hyperbolic materials (*ε*_‖_ < 0, *ε*_⊥_ > 0), and Type-II hyperbolic materials (*ε*_⊥_ < 0, *ε*_‖_ > 0), respectively.

The energy propagation direction of polaritons, defined by the angle *β* between the Poynting vector and *z*-axis, is defined by [29]:

(3) $$\beta \left(\omega \right)=\arctan\left(i\frac{\sqrt{\epsilon_{⊥}\left(\omega \right)}}{\sqrt{\epsilon_{\|}\left(\omega \right)}}\right).$$

In some cases, Equation (3) is expressed as [28,30]:

(4) $$\beta \left(\omega \right)=\frac{\pi }{2}-\arctan\left(\frac{\sqrt{\epsilon_{\|}\left(\omega \right)}}{i\sqrt{\epsilon_{⊥}\left(\omega \right)}}\right).$$

As shown in Figure 5 and Equations (3) and (4), these hyperbolic regions allow *k_x_* and *k_y_* to become infinitely large in hBN, enabling light to be folded within the material. This is the origin of the strong light confinement in extremely small volumes of hBN [28,31] and its ability to realize sub-diffraction imaging [29,32]. Such HPPs have been directly observed in both multilayer [33,34] and monolayer [35] hBN using near-field microscopy. Furthermore, inducing curvature in hBN structures has been shown to modify the HPPs [36,37]. Controlling the thickness (layer number) and shape of hBN is critical for the development of next-generation HPP-based photonic devices.

It should be noted that orthorhombic-phase molybdenum trioxide (α-MoO_3_), which is another vdW material, also exhibits HPPs in the IR region [38,39,40].

## 3. Ultraviolet Devices

Because the bandgap of hBN lies in the DUV region, hBN has excellent potential for the design of DUV-LEDs and DUV-PDs. These applications are discussed in this section.

### 3.1. DUV-LEDs

DUV-LEDs have attracted considerable attention for a wide range of applications, including sterilization, water purification, photocatalysis, and curing. In particular, interest in UV disinfection technologies has rapidly grown owing to the COVID-19 pandemic [41].

AlGaN semiconductors are the most widely investigated materials for fabricating DUV-LEDs [42]. hBN was initially introduced as an electron-blocking and p-contact layer to enhance the hole injection efficiency, reduce the contact resistance, and increase the UV transparency of AlGaN-based LEDs. However, AlGaN-based DUV-LEDs emit light perpendicular to the surface plane [43], whereas the light emitted from hBN is parallel to the surface plane. Furthermore, hBN can be formed on arbitrary substrates with high DUV transparency owing to the vdW forces of hBN. Therefore, hBN is considered an efficient emissive layer for use in DUV-LEDs.

Multilayer hBN can produce strong photoluminescence [22,44] and cathodoluminescence [20,21,44,45,46] in the DUV region despite its indirect bandgap. This phenomenon is attributed to the strong coupling of the exciton–phonon interactions [46]. Such phonon-assisted recombination is fast enough (~100 ps) to bypass non-radiative relaxation [44,47]. Consequently, the internal quantum efficiency is comparable to that of semiconductors with a direct bandgap [46]. Thus, hBN has the advantages of both direct and indirect bandgap semiconductors, which helps to achieve both a high extraction efficiency and internal quantum efficiency.

**Figure 6:**
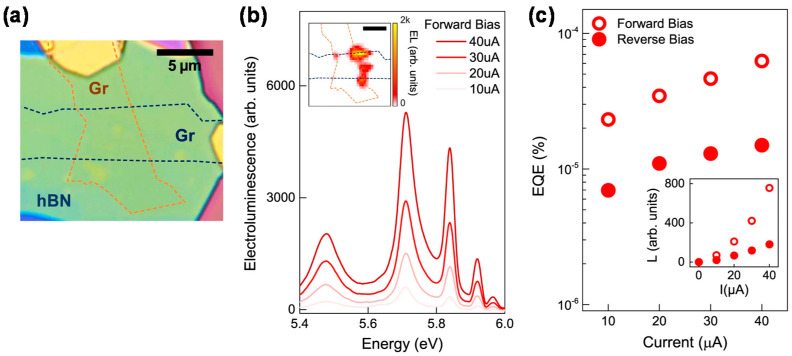
(**a**) Optical microscope image of the graphene/hBN/graphene van der Waals (vdW) heterostructure. (**b**) Deep ultraviolet (DUV) electroluminescence spectra under forward bias. (**c**) Dependence of the external quantum efficiency (EQE) of electroluminescence on current. Adapted from [48] under CC BY 4.0.

The electroluminescence of hBN in the DUV region has not been demonstrated thus far. Recently, the electroluminescence of a graphene/hBN/graphene vdW heterostructure was demonstrated at room temperature (Figure 6) [48]. The graphene/hBN/graphene vdW heterostructure (Figure 6a), in which the photoactive layer of hBN was sandwiched between a pair of graphene layers as electrodes, was fabricated from exfoliated graphene and hBN via a dry transfer technique. Carrier injection from the graphene electrodes to the hBN band edges occurred under high bias voltages via Fowler–Nordheim tunneling.

Figure 6b shows the electroluminescence spectra of the graphene/hBN/graphene vdW heterostructure under a forward bias at various currents, and Figure 6c shows the dependence of the electroluminescence external quantum efficiency (EQE) on the current under forward and reverse biases. The EQE gradually increased with the increasing current. The difference between the two bias polarities stems from asymmetric charge carrier injection owing to the asymmetric shape of the device. The obtained EQE was low; however, it could be improved by using a symmetric device structure with balanced carrier injection, a blocking layer, and multiple emissive layers. In particular, the formation of p–n junctions is important for achieving high-performance hBN-based DUV optoelectronics. p-type doping has been achieved by introducing Mg dopant atoms [49,50,51] or B vacancies [52]. Recently, n-type doping has also been demonstrated by Ge–O doping [53]. Along with other 2D materials, such technologies and suitable fabrication methods can help to realize highly efficient and stable DUV-LEDs.

### 3.2. DUV-PDs

Various UV-PDs have been developed from materials including AlGaN/GaN-based semiconductors [54,55], SiC-based semiconductors [56,57], oxide-based materials [58,59], and 2D materials [60] to cater to a wide range of applications, including advanced communications technologies, flare detection systems, and air purification devices [61].

Owing to its bandgap in the DUV region, various hBN-based UV-PDs have been developed [62,63,64]. Among them, hBN/Cu Schottky junctions and hBN/graphene vdW heterostructures have achieved substantially higher responsivities than single-hBN-based PDs. In particular, the photogating effect used in PDs based on hBN/graphene vdW heterostructures has recently drawn considerable research attention [65,66,67,68]. In these devices, hBN surrounds the graphene layer and acts as a photosensitizer by producing a voltage change upon light irradiation. Owing to the high carrier mobility and low atomic thickness of graphene, this voltage change can produce an extraordinary change in the carrier density of graphene [69]. As a result, the photocurrent can change significantly.

**Figure 7:**
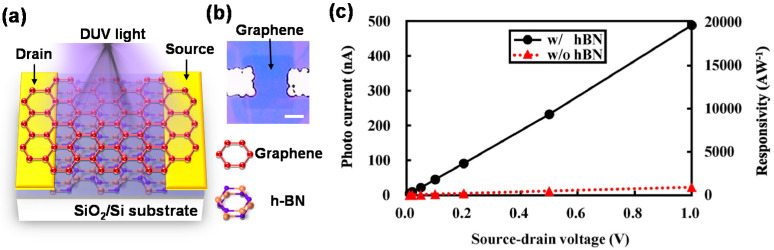
(**a**) Schematic and (**b**) optical microscopy image of hBN/graphene DUV photodetector (PD) prepared by photogating. Scale bar: 5 µm. (**c**) Photocurrent and responsivity as a function of source–drain voltage for DUV-PDs with and without hBN. Figures are redrawn with permission from [70]. Copyright 2022 Optica.

Figure 7a,b respectively show a schematic and image of a photogated hBN/graphene DUV-PD [70]. The PD structure is based on a simple graphene-based field effect transistor (GFETs). hBN is placed under the graphene channel and acts as a photosensitizer. It produces a back-gate voltage change in the GFET under DUV light irradiation, enabling the current in the graphene channel to be modulated substantially. Figure 7c shows the photo current and responsivity as a function of the source–drain voltage for DUV-PDs with and without hBN. The results demonstrate that ultra-high responsivity can be achieved by photogating.

As discussed in Section 3.1, the realization of hBN p–n junctions can facilitate the development of high-performance DUV-PDs, which cannot be achieved with conventional UV-PD technologies. In addition, the development of electrical readout circuits suitable for hBN would enable the fabrication of DUV-based image sensors.

## 4. Perfect Infrared Absorbers/Emitters

In addition to its performance in the DUV wavelength region, hBN has a distinguished potential for use in photonic devices operating in the infrared (IR) region. Metamaterial/metasurface-inspired or plasmonics-inspired “perfect” IR absorbers and emitters [71,72] have emerged as key materials for use in radiative cooling [73,74], advanced functional IR sensors [75], and biosensing [76] applications. hBN is a promising material for small and highly efficient absorbers and emitters because it is a natural hyperbolic material in the IR region, which allows photons to be confined within an ultra-small volume of hBN.

**Figure 8:**
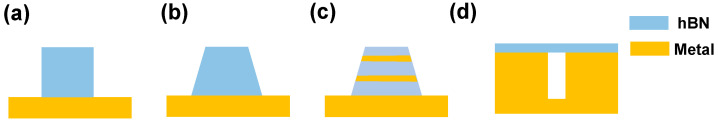
Unit cells of hBN-based metasurface absorbers/emitters (MAEs): (**a**) rectangular hBN/metal, (**b**) trapezoidal hBN/metal, (**c**) multilayer hBN/metal, and (**d**) hBN with plasmonic nanostructure grating.

The thermal radiation and absorbance of hBN can be controlled by adjusting the layer number [77]. Figure 8 shows unit cells of several hBN-based metasurface absorbers/emitters (hBN-MAEs) based on the well-known metal–insulator–metal (MIM) metasurface structure. Figure 8a shows the simplest hBN-MAE structure, in which rectangular blocks of hBN are placed periodically on a metal plate [78]. The most commonly used metals are Au, Ag, or Al because these metals support surface plasmon resonance (SPR). Figure 8b shows a slightly more complex structure, in which the sides of the hBN blocks are tapered [78]. Figure 8c shows an hBN-MAE in which a multilayer MIM pattern is introduced [79]. Finally, Figure 8d shows a structure in which hBN is placed on a metal grate [80,81,82]. Notably, devices with these structures have been shown to be promising IR absorbers/emitters.

**Figure 9:**
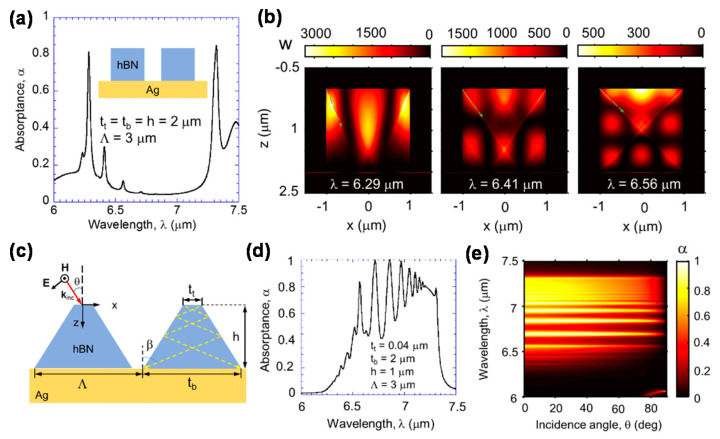
(**a**) Absorption spectrum of rectangular hBN-based MAE (inset: schematic) and (**b**) power dissipation contours at wavelengths of 6.29 μm (**left**), 6.41 μm (**middle**), and 6.56 μm (**right**). (**c**) Schematic of trapezoidal hBN, (**d**) absorption spectrum, and (**e**) absorption as a function of the incident angle and wavelength. Figures were adapted with permission from [78]. Copyright 2017 Optica.

Here, the basic absorption properties of hBN due to the HPPs are explained with consideration to the simplest hBN-MAE structure, that is, the rectangular hBN/metal structure (Figure 8a). Figure 9a shows a schematic of the device and its absorption spectrum. Figure 9b shows the power dissipation contours at wavelengths of 6.29, 6.41, and 6.56 μm, which correspond to the absorption peaks in the absorption spectrum (Figure 9a) in the Type-II hyperbolic region. Figure 9b clearly shows three zigzag resonant modes, demonstrating the strong confinement of light inside hBN owing to the HPPs. The resonance modes at wavelengths of 6.29, 6.41, and 6.56 μm are defined as (*m*, *n*) = (3, 1), (3, 2), and (3, 3), respectively.

Based on these resonance modes, the propagation direction can be described by [78]:

(5) $$\tan\beta =\frac{n}{m}.$$

Notably, this equation is important in the design of hBN-based photonic devices using HPPs. This is because the resonance modes are defined by the aspect ratio of the hBN cavity and not by its overall shape. This is a unique property of HPPs and is not observed with conventional resonance such as Fabry–Pérot resonance [28,83].

Figure 9c–e respectively show a schematic of the trapezoidal hBN/metal structure, its absorption spectrum, and its absorbance as a function of the incident angle and wavelength. As shown in Figure 9c, in the trapezoidal hBN resonator, *β* can be modified based on the taper angle, leading to broader absorption.

**Figure 10:**
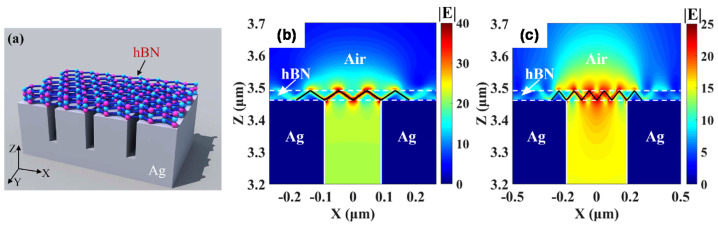
(**a**) Schematic of hBN on one-dimensional (1D) plasmonic grating. Electric field distribution of hBN on 1D plasmonic gratings with groove widths of (**b**) 180 and (**c**) 360 nm. Figures were adapted with permission from [81]. Copyright 2020 Optica.

The combination of SPR and HPPs can enhance the unique absorption properties of hBN. The multilayer hBN/metal structure shown in Figure 8c can produce broadband absorption owing to the SPR induced by the metal and the resulting hybridization of HPPs [79]. In addition, when hBN is placed on a Ag-based one-dimensional (1D) plasmonic grating, as shown schematically in Figure 8d and Figure 10a, it exhibits enhanced absorption due to coupling between the HPPs of hBN and the SPR or magnetic polaritons induced by the 1D plasmonic grating [84,85,86]. Figure 10b,c show the electric field distributions of hBN on 1D plasmonic gratings with different groove widths. Zigzag propagation modes were induced within the hBN layer owing to coupling between the magnetic polaritons induced by the 1D plasmonic grating and the HPPs of hBN. Because the hyperbolic properties follow Equations (3) or (4), the change in coupling frequency with groove width is minimal.

Plasmonic structures such as 2D plasmonic crystals and asymmetric structures [87,88] can be used to enhance the interaction between SPR induced by the plasmonic structure and the HPPs in hBN. This enhanced interaction can facilitate the design of novel photonic devices that employ IR absorption and emission. In addition to the aforementioned IR absorbers/emitters, hBN is frequently used in graphene-based metamaterial IR absorbers/emitters [89,90,91,92,93]; the use of patterned graphene can result in strong absorption induced by localized SPR in the IR region [94,95].

## 5. Sub-Diffraction-Limit Imaging

Hyperbolic materials allow large wavevector (*k*) values. In hyperlenses, the light scattered from sub-diffraction-limit features is coupled with the HPPs, which propagates the light in the expanded direction according to Equation (3). As a result, sub-diffraction-limit imaging can be achieved.

Early research into hyperlenses used hyperbolic metamaterials (HMMs) with artificially engineered structures [96,97] to demonstrate hyperlenses or superlenses [98,99,100]. However, conventional HMMs require complicated artificial structures such as multilayers of dielectric and metal films. Furthermore, metal films induce optical loss for imaging. Therefore, hyperlenses using HMMs suffer from a low transmission efficiency and limited spatial resolution [101,102]. On the contrary, hBN is a natural hyperbolic material with low-loss HPPs in two RS bands. Therefore, hBN is suitable for the development of high-performance hyperlenses.

**Figure 11:**
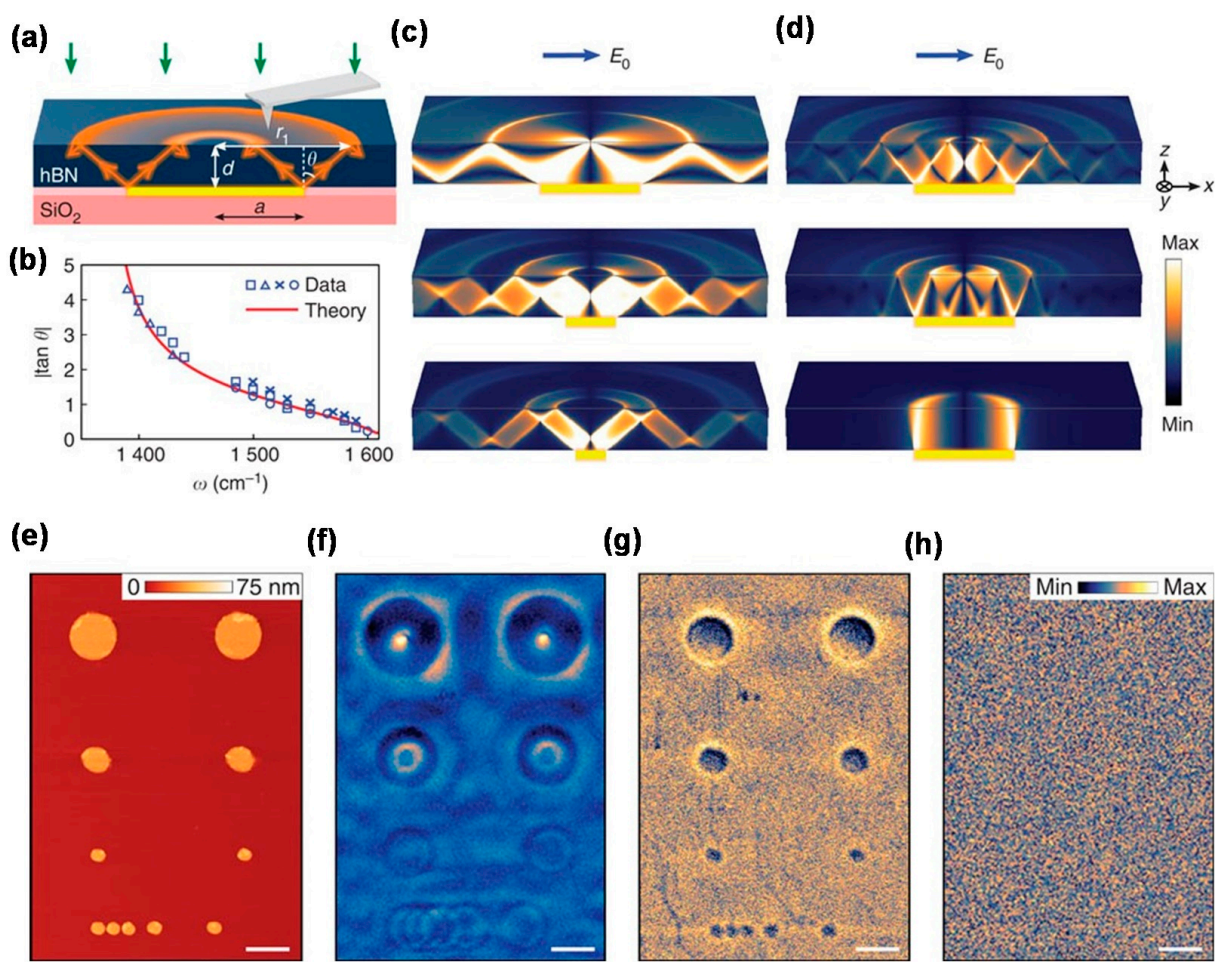
(**a**) Concept of a hBN-based hyperlens. The yellow rectangle shows the Au disk. (**b**) |tan*θ*| as a function of frequency. (**c**,**d**) Electric field distribution in the *z* direction with (**c**) *a*/*d* = 0.5 (**top**), 0.25 (**middle**), and 0.15 (**bottom**) and (**d**) *a*/*d* = 1.12 and |tan*θ*| = 0.75 (**top**), 0.375 (**middle**), and 0.01 (**bottom**). (**e**) Atomic force microscopy (AFM) image of Au disks. Scattering-type scanning near-field optical microscopy (s-SNOM) images at infrared (IR) laser frequencies ω of (**f**) 1515 cm^−1^ (*λ* = 6.6 μm), (**g**) 1610 cm^−1^ (*λ* = 6.2 μm), and (**h**) 1740 cm^−1^ (*λ* = 5.7 μm). Figures were adapted from [29] under CC BY 4.0.

A schematic of a hBN-based hyperlens is shown in Figure 11a. The hyperlens was demonstrated with Au disks embedded in hBN [29,32] and visualized by scattering-type scanning near-field optical microscopy (s-SNOM). HPPs propagate light from the edges of the Au disks under IR illumination. Figure 11c,d show the electric field distribution in the *z* direction for hBN-based hyperlenses with different aspect ratios (*a*/*d*, where *d* is the thickness of the hBN film and *a* is the radius of the Au disk) and propagation directions (*θ*) [29]. Here, *θ* corresponds to *β* in Equations (3) and (4). These calculated results demonstrate that the unique zigzag propagation of the HPPs enables the hyperlens behavior and the aspect ratio *a*/*d* defines the propagation mode. Figure 11e shows an atomic force microscopy (AFM) image of the Au nanodisks and Figure 11f–h show s-SNOM images of the Au nanodisks taken at IR laser frequencies within, at the boundary of, and outside the hyperbolic region, respectively. These figures clearly show that sub-diffraction-limit imaging with a resolution of *λ*/33 was achieved in the Type-II hyperbolic region.

hBN-based hyperlenses have been used for various applications, including in defect diagnosis [103]. Furthermore, compared with naturally abundant hBN, the use of monoisotopic h^11^BN (>99%) [104] has been shown to reduce optical losses by a factor of three and an imaging resolution of at least *λ*/154 was achieved [102]. Moreover, a graphene/hBN multilayer hypercrystal achieved high-efficiency negative transmission, which can promote superlens imaging at high resolutions. Therefore, hBN-based hyperlenses have promising applications in biomedical imaging, internal structure diagnosis, label-free detection, and fine-structure analysis.

## 6. HPP-Based Sensors

IR spectroscopy is a powerful analytical tool for label-free and non-destructive material characterization based on the unique fingerprints of molecules. In particular, surface-enhanced IR absorption (SEIRA) spectroscopy is promising for high-precision material analysis [105]. SEIRA spectroscopy is based on the strong interaction between IR light and molecules in the strongly confined near field of localized SPRs (LSPRs). However, in conventional SEIRA spectroscopy, noble metals such as Au are used to produce LSPRs, which inevitably involves losses, and the quality factor (*Q*) therein is limited to ~10 [106]. In addition, although graphene-based LSPRs can be tuned by doping, the resultant *Q* is poor [107].

**Figure 12:**
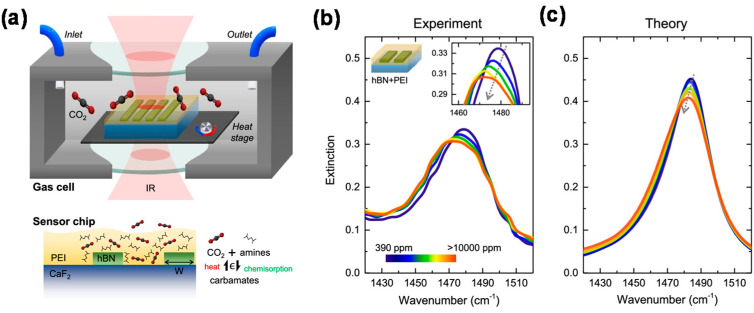
(**a**) Schematic of the CO_2_ gas sensing setup and sensor chip using hBN nanoribbons. Extinction spectra for CO_2_: (**b**) experimental and (**c**) theoretical results. Adapted from [109] under CC BY 4.0.

In contrast, because the HPPs of hBN can confine IR light to a small cavity, hBN can achieve a high *Q* of ~100 [104]. Therefore, hBN is a strong candidate for SEIRA spectroscopy. 4,4′-Bis(*N*-carbazolyl)-1,1′-biphenyl and CO_2_ sensing using hBN nanoribbons have been demonstrated previously [108,109]. Figure 12a schematically shows the CO_2_ gas sensing setup and cross-section of the CO_2_-sensing chip using hBN nanoribbons. In this device, the blueshift of the HPPs in the Type-II hyperbolic region increases with increasing CO_2_ concentration, as shown in Figure 12b,c, thereby enabling CO_2_ sensing [109].

For advanced SEIRA spectroscopy, hBN with tunable HPPs based on graphene and a 1D plasmonic grating has been proposed [82]. The electrical gating enables control over the Fermi level of graphene, which thus provides electrical tunability to hBN. The high *Q* of high-quality hBN and electrical tunability of graphene could contribute to the development of next-generation biosensing devices.

## 7. Future Outlook

hBN has excellent potential for use in photonic devices that operate in both the DUV and IR wavelength regions owing to its unique bandgap structure and the hyperbolicity of its dielectric constant. In addition to the applications discussed in this review, various other applications of hBN-based photonic devices have been proposed, including ultra-thin wavelength-selective filters [110], waveguides [111,112,113,114], and photonic crystals [115].

The rapid progress in the fabrication of hBN-based p–n junctions is expected to enable the development of high-performance DUV-LEDs and DUV-PDs in the near future. In the IR wavelength region, the artificial control of HPPs is crucial for the development of novel photonic devices, including thermal control devices [116,117]. Engineered surfaces such as metasurfaces [118,119,120] and waveguides [121] are crucial for controlling the HPPs. Graphene/hBN hybrids can afford HPP tunability for tunable IR photonic devices [26,122,123]. Moreover, the concept of topological photonics [124] is expected to broaden the field of HPP-based photonic devices.

Stacking layer angle control, as in “twistronics,” is a unique method of bandgap structure engineering that can produce novel properties, such as DUV emission band modification [125], ferroelectricity [126], and color center tunability [127]. Furthermore, similar to turbostratic graphene, turbostratic hBN is expected to exhibit the properties of monolayer hBN even in multilayer structures [128,129].

The crucial issues pertaining to the development of hBN-based photonic devices include the fabrication of hBN, its transfer to an arbitrary substrate, and its compatibility with complementary metal-oxide semiconductor (CMOS) technologies [130,131,132]. These issues are also commonly encountered for other 2D materials used to fabricate electric and photonic devices. Owing to the difficulty in fabricating high-quality hBN films, the excellent properties of hBN- and hBN/graphene-based devices have been demonstrated using exfoliated hBN. Therefore, it is crucial to develop mass fabrication technologies for the production of high-quality hBN films, such as by using chemical vapor deposition (CVD) and transferring the prepared film onto an arbitrary substrate. Among the various CVD techniques [133,134,135], the seamless CVD fabrication of graphene/hBN interfaces is the most important [136,137], because wet transfer processes inevitably introduce contamination at the hBN/graphene interface. This contamination degrades the high carrier mobility of graphene, even with a high-quality hBN film. Superior etching techniques are also needed, because conventional etching techniques degrade the quality of hBN [138], thereby hindering the development of high-performance hBN-based photonic devices. Recent progress in the fabrication of 2D materials is expected to enable the production of high-quality hBN that could be compatible with CMOS technologies.

## 8. Conclusions

Herein, we reviewed the applications of hBN-based photonic devices based on two unique properties of hBN, namely, the indirect bandgap in the DUV wavelength region and the HPPs in the IR wavelength region. The development of DUV-LEDs and DUV-PDs, which exploit the unique indirect bandgap structure of hBN, is in the initial stage. However, p- and n-type doping and photogating effects can considerably improve their performance. The HPPs of hBN have been employed in numerous applications, including IR absorbers/emitters, hyperlenses, and SEIRA sensing devices. The HPPs enable IR light to be confined to ultra-small volumes within hBN, which facilitates wavelength-selective and broadband absorption and emission. The HPPs enable hBN to be employed in the development of hyperlenses owing to the large *k* vector. The strong localized fields of HPPs result in a high *Q* value, which facilitates SEIRA spectroscopy for molecule or gas sensing. Given the importance of hBN, its fabrication methods, such as CVD, must be drastically improved. A breakthrough in the fabrication technologies is expected to enable the mass production of high-performance hBN-based photonic devices. Photonic devices operating in the UV and IR wavelength regions have growing applications in security and everyday life. This review is expected to contribute to the development of hBN-based photonic devices and widen their applications.

## Data Availability

No new data were created or analyzed in this study.

## References

[1] Ben J., Liu X., Wang C., Zhang Y., Shi Z., Jia Y., Zhang S., Zhang H., Yu W., Li D. (2021). 2D III-Nitride Materials: Properties, Growth, and Applications. Adv. Mater..

[2] Merlo A., Mokkapati V.R.S.S., Pandit S., Mijakovic I. (2018). Boron nitride nanomaterials: Biocompatibility and bio-applications. Biomater. Sci..

[3] Dean C.R., Young A.F., Meric I., Lee C., Wang L., Sorgenfrei S., Watanabe K., Taniguchi T., Kim P., Shepard K.L. (2010). Boron nitride substrates for high-quality graphene electronics. Nat. Nanotechnol..

[4] Woessner A., Lundeberg M.B., Gao Y., Principi A., Alonso-González P., Carrega M., Watanabe K., Taniguchi T., Vignale G., Polini M. (2015). Highly confined low-loss plasmons in graphene–boron nitride heterostructures. Nat. Mater..

[5] Wang J., Ma F., Liang W., Wang R., Sun M. (2017). Optical, photonic and optoelectronic properties of graphene, h-BN and their hybrid materials. Nanophotonics.

[6] Caldwell J.D., Aharonovich I., Cassabois G., Edgar J.H., Gil B., Basov D.N. (2019). Photonics with hexagonal boron nitride. Nat. Rev. Mater..

[7] Li L.H., Chen Y. (2016). Atomically Thin Boron Nitride: Unique Properties and Applications. Adv. Func. Mater..

[8] Low T., Chaves A., Caldwell J.D., Kumar A., Fang N.X., Avouris P., Heinz T.F., Guinea F., Martin-Moreno L., Koppens F. (2017). Polaritons in layered two-dimensional materials. Nat. Mater..

[9] Revabhai P.M., Singhal R.K., Basu H., Kailasa S.K. (2022). Progress on boron nitride nanostructure materials: Properties, synthesis and applications in hydrogen storage and analytical chemistry. J. Nanostruct. Chem..

[10] Maestre C., Toury B., Steyer P., Garnier V., Journet C. (2021). Hexagonal boron nitride: A review on selfstanding crystals synthesis towards 2D nanosheets. J. Phys. Mater..

[11] Geim A.K., Grigorieva I.V. (2013). Van der Waals heterostructures. Nature.

[12] Novoselov K.S., Mishchenko A., Carvalho A., Castro Neto A.H. (2016). 2D materials and van der Waals heterostructures. Science.

[13] Shaik A.B.D., Palla P. (2021). Optical quantum technologies with hexagonal boron nitride single photon sources. Sci. Rep..

[14] Kubanek A. (2022). Coherent Quantum Emitters in Hexagonal Boron Nitride. Adv. Quantum Technol..

[15] Xu Y.N., Ching W.Y. (1991). Calculation of ground-state and optical properties of boron nitrides in the hexagonal, cubic, and wurtzite structures. Phys. Rev. B.

[16] Furthmüller J., Hafner J., Kresse G. (1994). Ab initio calculation of the structural and electronic properties of carbon and boron nitride using ultrasoft pseudopotentials. Phys. Rev. B.

[17] Blase X., Rubio A., Louie S.G., Cohen M.L. (1995). Quasiparticle band structure of bulk hexagonal boron nitride and related systems. Phys. Rev. B.

[18] Arnaud B., Lebègue S., Rabiller P., Alouani M. (2006). Huge excitonic effects in layered hexagonal boron nitride. Phys. Rev. Lett..

[19] Zunger A., Katzir A., Halperin A. (1976). Optical properties of hexagonal boron nitride. Phys. Rev. B.

[20] Watanabe K., Taniguchi T., Kanda H. (2004). Direct-bandgap properties and evidence for ultraviolet lasing of hexagonal boron nitride single crystal. Nat. Mater..

[21] Kubota Y., Watanabe K., Tsuda O., Taniguchi T. (2007). Deep ultraviolet light-emitting hexagonal boron nitride synthesized at atmospheric pressure. Science.

[22] Cassabois G., Valvin P., Gil B. (2016). Hexagonal boron nitride is an indirect bandgap semiconductor. Nat. Photon..

[23] Watanabe K., Taniguchi T. (2009). Jahn-Teller effect on exciton states in hexagonal boron nitride single crystal. Phys. Rev. B.

[24] Wickramaratne D., Weston L., Van de Walle C.G. (2018). Monolayer to Bulk Properties of Hexagonal Boron Nitride. J. Phys. Chem. C.

[25] Ferreira F., Chaves A.J., Peres N.M.R., Ribeiro R.M. (2019). Excitons in hexagonal boron nitride single-layer: A new platform for polaritonics in the ultraviolet. J. Opt. Soc. Am. B.

[26] Kumar A., Low T., Fung K.H., Avouris P., Fang N.X. (2015). Tunable Light-Matter Interaction and the Role of Hyperbolicity in Graphene-hBN System. Nano Lett..

[27] Cai Y., Zhang L., Zeng Q., Cheng L., Xu Y. (2007). Infrared reflectance spectrum of BN calculated from first principles. Solid State Commun..

[28] Caldwell J.D., Kretinin A.V., Chen Y., Giannini V., Fogler M.M., Francescato Y., Ellis C.T., Tischler J.G., Woods C.R., Giles A.J. (2014). Sub-diffractional volume-confined polaritons in the natural hyperbolic material hexagonal boron nitride. Nat. Commun..

[29] Dai S., Ma Q., Andersen T., McLeod A.S., Fei Z., Liu M.K., Wagner M., Watanabe K., Taniguchi T., Thiemens M. (2015). Subdiffractional focusing and guiding of polaritonic rays in a natural hyperbolic material. Nat. Commun..

[30] Giles A.J., Dai S., Glembocki O.J., Kretinin A.V., Sun Z., Ellis C.T., Tischler J.G., Taniguchi T., Watanabe K., Fogler M.M. (2016). Imaging of Anomalous Internal Reflections of Hyperbolic Phonon-Polaritons in Hexagonal Boron Nitride. Nano Lett..

[31] Tamagnone M., Ambrosio A., Chaudhary K., Jauregui L.A., Kim P., Wilson W.L., Capasso F. (2018). Ultra-confined mid-infrared resonant phonon polaritons in van der Waals nanostructures. Sci. Adv..

[32] Li P., Lewin M., Kretinin A.V., Caldwell J.D., Novoselov K.S., Taniguchi T., Watanabe K., Gaussmann F., Taubner T. (2015). Hyperbolic phonon-polaritons in boron nitride for near-field optical imaging and focusing. Nat. Commun..

[33] Dai S., Fei Z., Ma Q., Rodin A.S., Wagner M., McLeod A.S., Liu M.K., Gannett W., Regan W., Watanabe K. (2014). Tunable Phonon Polaritons in Atomically Thin van der Waals Crystals of Boron Nitride. Science.

[34] Dai S., Tymchenko M., Yang Y., Ma Q., Pita-Vidal M., Watanabe K., Taniguchi T., Jarillo-Herrero P., Fogler M.M., Alu A. (2018). Manipulation and Steering of Hyperbolic Surface Polaritons in Hexagonal Boron Nitride. Adv. Mater..

[35] Dai S., Fang W., Rivera N., Stehle Y., Jiang B.Y., Shen J., Tay R.Y., Ciccarino C.J., Ma Q., Rodan-Legrain D. (2019). Phonon Polaritons in Monolayers of Hexagonal Boron Nitride. Adv. Mater..

[36] Zhou Y., Qi D.X., Wang Y.K. (2017). Phonon polaritons in cylindrically curved h-BN. Opt. Express.

[37] Yan X., Li J., Gu L., Gadre C.A., Moore S.L., Aoki T., Wang S., Zhang G., Gao Z., Basov D.N. (2022). Curvature-Induced One-Dimensional Phonon Polaritons at Edges of Folded Boron Nitride Sheets. Nano Lett..

[38] Ma W., Alonso-González P., Li S., Nikitin A.Y., Yuan J., Martín-Sánchez J., Taboada-Gutiérrez J., Amenabar I., Li P., Vélez S. (2018). In-plane anisotropic and ultra-low-loss polaritons in a natural van der Waals crystal. Nature.

[39] Zheng Z., Chen J., Wang Y., Wang X., Chen X., Liu P., Xu J., Xie W., Chen H., Deng S. (2018). Highly Confined and Tunable Hyperbolic Phonon Polaritons in Van Der Waals Semiconducting Transition Metal Oxides. Adv. Mater..

[40] Zheng Z., Xu N., Oscurato S.L., Tamagnone M., Sun F., Jiang Y., Ke Y., Chen J., Huang W., Wilson W.L. (2019). A mid-infrared biaxial hyperbolic van der Waals crystal. Sci. Adv..

[41] Raeiszadeh M., Adeli B. (2020). A Critical Review on Ultraviolet Disinfection Systems against COVID-19 Outbreak: Applicability, Validation, and Safety Considerations. ACS Photonics.

[42] Mondal R.K., Adhikari S., Chatterjee V., Pal S. (2021). Recent advances and challenges in AlGaN-based ultra-violet light emitting diode technologies. Mater. Res. Bull..

[43] Jiang H.X., Lin J.Y. (2014). Hexagonal boron nitride for deep ultraviolet photonic devices. Semicond. Sci. Technol..

[44] Watanabe K., Taniguchi T., Miya K., Sato Y., Nakamura K., Niiyama T., Taniguchi M. (2011). Hexagonal boron nitride as a new ultraviolet luminescent material and its application—Fluorescence properties of hBN single-crystal powder. Diam. Relat. Mater..

[45] Pierret A., Loayza J., Berini B., Betz A., Plaçais B., Ducastelle F., Barjon J., Loiseau A. (2014). Excitonic recombinations in h−BN: From bulk to exfoliated layers. Phys. Rev. B.

[46] Schué L., Sponza L., Plaud A., Bensalah H., Watanabe K., Taniguchi T., Ducastelle F., Loiseau A., Barjon J. (2019). Bright Luminescence from Indirect and Strongly Bound Excitons in h-BN. Phys. Rev. Lett..

[47] Cassabois G., Valvin P., Gil B. (2016). Intervalley scattering in hexagonal boron nitride. Phys. Rev. B.

[48] Song S.B., Yoon S., Kim S.Y., Yang S., Seo S.Y., Cha S., Jeong H.W., Watanabe K., Taniguchi T., Lee G.H. (2021). Deep-ultraviolet electroluminescence and photocurrent generation in graphene/hBN/graphene heterostructures. Nat. Commun..

[49] Dahal R., Li J., Majety S., Pantha B.N., Cao X.K., Lin J.Y., Jiang H.X. (2011). Epitaxially grown semiconducting hexagonal boron nitride as a deep ultraviolet photonic material. Appl. Phys. Lett..

[50] Sun F., Hao Z., Liu G., Wu C., Lu S., Huang S., Liu C., Hong Q., Chen X., Cai D. (2018). p-Type conductivity of hexagonal boron nitride as a dielectrically tunable monolayer: Modulation doping with magnesium. Nanoscale.

[51] Wang Y., Liu G., Lu S., Zhang H., Guo B., Du G., Chen X., Cai D., Kang J. (2020). Enhancement of p-type conductivity of monolayer hexagonal boron nitride by driving Mg incorporation through low-energy path with N-rich condition. Appl. Phys. Lett..

[52] Laleyan D.A., Zhao S., Woo S.Y., Tran H.N., Le H.B., Szkopek T., Guo H., Botton G.A., Mi Z. (2017). AlN/h-BN Heterostructures for Mg Dopant-Free Deep Ultraviolet Photonics. Nano Lett..

[53] Lu S., Shen P., Zhang H., Liu G., Guo B., Cai Y., Chen H., Xu F., Zheng T., Xu F. (2022). Towards n-type conductivity in hexagonal boron nitride. Nat. Commun..

[54] Tan R., Cai Q., Wang J., Pan D., Li Z., Chen D. (2021). Highly solar-blind ultraviolet selective metal-semiconductor-metal photodetector based on back-illuminated AlGaN heterostructure with integrated photonic crystal filter. Appl. Phys. Lett..

[55] Jiang K., Sun X., Chen Y., Zhang S., Ben J., Chen Y., Zhang Z.-H., Jia Y., Shi Z., Li D. (2021). Three-dimensional metal–semiconductor–metal bipolar ultraviolet phototransistor based on GaN p-i-n epilayer. Appl. Phys. Lett..

[56] Chen X., Zhu H., Cai J., Wu Z. (2007). High-performance 4H-SiC-based ultraviolet p-i-n photodetector. J. Appl. Phys..

[57] Aldalbahi A., Li E., Rivera M., Velazquez R., Altalhi T., Peng X., Feng P.X. (2016). A new approach for fabrications of SiC based photodetectors. Sci. Rep..

[58] Kim S., Kim J. (2020). Highly selective ozone-treated β-Ga2O3 solar-blind deep-UV photodetectors. Appl. Phys. Lett..

[59] Han D., Liu K., Chen X., Li B., Zhai T., Liu L., Shen D. (2021). Performance enhancement of a self-powered solar-blind UV photodetector based on ZnGa2O4/Si heterojunction via interface pyroelectric effect. Appl. Phys. Lett..

[60] Yu Y., Shen T., Long H., Zhong M., Xin K., Zhou Z., Wang X., Liu Y.Y., Wakabayashi H., Liu L. (2022). Doping Engineering in the MoS(2) /SnSe(2) Heterostructure toward High-Rejection-Ratio Solar-Blind UV Photodetection. Adv. Mater..

[61] Chen H., Liu K., Hu L., Al-Ghamdi A.A., Fang X. (2015). New concept ultraviolet photodetectors. Mater. Today.

[62] Zhou A.F., Aldalbahi A., Feng P. (2016). Vertical metal-semiconductor-metal deep UV photodetectors based on hexagonal boron nitride nanosheets prepared by laser plasma deposition. Opt. Mater. Express.

[63] Veeralingam S., Durai L., Yadav P., Badhulika S. (2021). Record-High Responsivity and Detectivity of a Flexible Deep-Ultraviolet Photodetector Based on Solid State-Assisted Synthesized hBN Nanosheets. ACS Appl. Electron. Mater..

[64] Yang H., Wang L., Gao F., Dai M., Hu Y., Chen H., Zhang J., Qiu Y., Jia C., Zhou Y. (2019). Shape evolution of two dimensional hexagonal boron nitride single domains on Cu/Ni alloy and its applications in ultraviolet detection. Nanotechnology.

[65] Shimatani M., Ogawa S., Fujisawa D., Okuda S., Kanai Y., Ono T., Matsumoto K. (2016). Giant Dirac point shift of graphene phototransistors by doped silicon substrate current. AIP Adv..

[66] Kufer D., Konstantatos G. (2016). Photo-FETs: Phototransistors Enabled by 2D and 0D Nanomaterials. ACS Photonics.

[67] Guo X., Wang W., Nan H., Yu Y., Jiang J., Zhao W., Li J., Zafar Z., Xiang N., Ni Z. (2016). High-performance graphene photodetector using interfacial gating. Optica.

[68] Shimatani M., Fukushima S., Okuda S., Ogawa S. (2020). High-performance graphene/InSb heterojunction photodetectors for high-resolution mid-infrared image sensors. Appl. Phys. Lett..

[69] Ogawa S., Shimatani M., Fukushima S., Okuda S., Kanai Y., Ono T., Matsumoto K. (2019). Broadband photoresponse of graphene photodetector from visible to long-wavelength infrared wavelengths. Opt. Eng..

[70] Fukushima S., Fukamachi S., Shimatani M., Kawahara K., Ago H., Ogawa S. (2022). Graphene-based deep-ultraviolet photodetectors with ultrahigh responsivity using chemical vapor deposition of hexagonal boron nitride to achieve photogating. Opt. Mater. Express.

[71] Watts C.M., Liu X., Padilla W.J. (2012). Metamaterial electromagnetic wave absorbers. Adv. Mater..

[72] Ogawa S., Kimata M. (2018). Metal-Insulator-Metal-Based Plasmonic Metamaterial Absorbers at Visible and Infrared Wavelengths: A Review. Materials.

[73] Zhai Y., Ma Y., David S.N., Zhao D., Lou R., Tan G., Yang R., Yin X. (2017). Scalable-manufactured randomized glass-polymer hybrid metamaterial for daytime radiative cooling. Science.

[74] Ko B., Lee D., Badloe T., Rho J. (2018). Metamaterial-Based Radiative Cooling: Towards Energy-Free All-Day Cooling. Energies.

[75] Ogawa S., Kimata M. (2017). Wavelength- or Polarization-Selective Thermal Infrared Detectors for Multi-Color or Polarimetric Imaging Using Plasmonics and Metamaterials. Materials.

[76] Liu N., Mesch M., Weiss T., Hentschel M., Giessen H. (2010). Infrared Perfect Absorber and Its Application as Plasmonic Sensor. Nano Lett..

[77] Zhao B., Song J.-H., Brongersma M., Fan S. (2021). Atomic-Scale Control of Coherent Thermal Radiation. ACS Photonics.

[78] Zhao B., Zhang Z.M. (2017). Resonance perfect absorption by exciting hyperbolic phonon polaritons in 1D hBN gratings. Opt. Express.

[79] Kan Y.H., Zhao C.Y., Zhang Z.M. (2018). Compact mid-infrared broadband absorber based on hBN/metal metasurface. Int. J. Therm. Sci..

[80] Zhao B., Zhang Z.M. (2017). Perfect mid-infrared absorption by hybrid phonon-plasmon polaritons in hBN/metal-grating anisotropic structures. Int. J. Heat Mass Transf..

[81] Hu J., Xie W., Chen J., Zhou L., Liu W., Li D., Zhan Q. (2020). Strong hyperbolic-magnetic polaritons coupling in an hBN/Ag-grating heterostructure. Opt. Express.

[82] Kumari R., Yadav A., Sharma S., Das Gupta T., Varshney S.K., Lahiri B. (2021). Tunable Van der Waal’s optical metasurfaces (VOMs) for biosensing of multiple analytes. Opt. Express.

[83] Sun Z., Gutiérrez-Rubio Á., Basov D.N., Fogler M.M. (2015). Hamiltonian Optics of Hyperbolic Polaritons in Nanogranules. Nano Lett..

[84] Miyazaki H.T., Ikeda K., Kasaya T., Yamamoto K., Inoue Y., Fujimura K., Kanakugi T., Okada M., Hatade K., Kitagawa S. (2008). Thermal emission of two-color polarized infrared waves from integrated plasmon cavities. Appl. Phys. Lett..

[85] Bouchon P., Pardo F., Portier B., Ferlazzo L., Ghenuche P., Dagher G., Dupuis C., Bardou N., Haïdar R., Pelouard J.-L. (2011). Total funneling of light in high aspect ratio plasmonic nanoresonators. Appl. Phys. Lett..

[86] Ogawa S., Kimata M. (2017). Direct fabrication and characterization of high-aspect-ratio plasmonic nanogratings using tapered-sidewall molds. Opt. Mater. Express.

[87] Ogawa S., Okada K., Fukushima N., Kimata M. (2012). Wavelength selective uncooled infrared sensor by plasmonics. Appl. Phys. Lett..

[88] Ogawa S., Takagawa Y., Kimata M. (2016). Fano resonance in asymmetric-period two-dimensional plasmonic absorbers for dual-band uncooled infrared sensors. Opt. Eng..

[89] Cai Y., Zhu J., Liu Q.H. (2015). Tunable enhanced optical absorption of graphene using plasmonic perfect absorbers. Appl. Phys. Lett..

[90] Wu J., Jiang L., Guo J., Dai X., Xiang Y., Wen S. (2016). Tunable perfect absorption at infrared frequencies by a graphene-hBN hyper crystal. Opt. Express.

[91] Hajian H., Ghobadi A., Butun B., Ozbay E. (2018). Tunable, omnidirectional, and nearly perfect resonant absorptions by a graphene-hBN-based hole array metamaterial. Opt. Express.

[92] Deng G., Song X., Dereshgi S.A., Xu H., Aydin K. (2019). Tunable multi-wavelength absorption in mid-IR region based on a hybrid patterned graphene-hBN structure. Opt. Express.

[93] Ogawa S., Fukushima S., Shimatani M. (2022). Hexagonal-boron nitride/graphene van der Waals heterostructure-based wavelength-selective infrared absorbers using plasmonic metasurfaces for multi-spectral infrared photodetectors. J. Opt. Soc. Am. B.

[94] García de Abajo F.J. (2014). Graphene Plasmonics: Challenges and Opportunities. ACS Photonics.

[95] Ogawa S., Fukushima S., Shimatani M. (2020). Graphene Plasmonics in Sensor Applications: A Review. Sensors.

[96] Poddubny A., Iorsh I., Belov P., Kivshar Y. (2013). Hyperbolic metamaterials. Nat. Photonics.

[97] Takayama O., Lavrinenko A.V. (2019). Optics with hyperbolic materials [Invited]. J. Opt. Soc. Am. B.

[98] Liu Z., Lee H., Xiong Y., Sun C., Zhang X. (2007). Far-field optical hyperlens magnifying sub-diffraction-limited objects. Science.

[99] Rho J., Ye Z., Xiong Y., Yin X., Liu Z., Choi H., Bartal G., Zhang X. (2010). Spherical hyperlens for two-dimensional sub-diffractional imaging at visible frequencies. Nat. Commun..

[100] Kawata S., Inouye Y., Verma P. (2009). Plasmonics for near-field nano-imaging and superlensing. Nat. Photonics.

[101] Lee D., Kim Y.D., Kim M., So S., Choi H.-J., Mun J., Nguyen D.M., Badloe T., Ok J.G., Kim K. (2018). Realization of Wafer-Scale Hyperlens Device for Sub-diffractional Biomolecular Imaging. ACS Photonics.

[102] He M., Iyer G.R.S., Aarav S., Sunku S.S., Giles A.J., Folland T.G., Sharac N., Sun X., Matson J., Liu S. (2021). Ultrahigh-Resolution, Label-Free Hyperlens Imaging in the Mid-IR. Nano Lett..

[103] Dai S., Tymchenko M., Xu Z.-Q., Tran T.T., Yang Y., Ma Q., Watanabe K., Taniguchi T., Jarillo-Herrero P., Aharonovich I. (2018). Internal Nanostructure Diagnosis with Hyperbolic Phonon Polaritons in Hexagonal Boron Nitride. Nano Lett..

[104] Giles A.J., Dai S., Vurgaftman I., Hoffman T., Liu S., Lindsay L., Ellis C.T., Assefa N., Chatzakis I., Reinecke T.L. (2018). Ultralow-loss polaritons in isotopically pure boron nitride. Nat. Mater..

[105] Wang H.L., You E.M., Panneerselvam R., Ding S.Y., Tian Z.Q. (2021). Advances of surface-enhanced Raman and IR spectroscopies: From nano/microstructures to macro-optical design. Light Sci. Appl..

[106] Lilley G., Messner M., Unterrainer K. (2015). Improving the quality factor of the localized surface plasmon resonance. Opt. Mater. Express.

[107] Rodrigo D., Limaj O., Janner D., Etezadi D., Abajo F.J.G.d., Pruneri V., Altug H. (2015). Mid-infrared plasmonic biosensing with graphene. Science.

[108] Autore M., Li P., Dolado I., Alfaro-Mozaz F.J., Esteban R., Atxabal A., Casanova F., Hueso L.E., Alonso-González P., Aizpurua J. (2018). Boron nitride nanoresonators for phonon-enhanced molecular vibrational spectroscopy at the strong coupling limit. Light Sci. Appl..

[109] Bareza N., Paulillo B., Slipchenko T.M., Autore M., Dolado I., Liu S., Edgar J.H., Vélez S., Martín-Moreno L., Hillenbrand R. (2022). Phonon-Enhanced Mid-Infrared CO2 Gas Sensing Using Boron Nitride Nanoresonators. ACS Photonics.

[110] Ogawa S., Fukushima S., Shimatani M. (2021). Extraordinary Optical Transmission by Hybrid Phonon-Plasmon Polaritons Using hBN Embedded in Plasmonic Nanoslits. Nanomaterials.

[111] Zhu B., Ren G., Zheng S., Lin Z., Jian S. (2013). Nanoscale dielectric-graphene-dielectric tunable infrared waveguide with ultrahigh refractive indices. Opt. Express.

[112] Zhu B., Ren G., Gao Y., Li H., Wu B., Jian S. (2016). Strong light confinement and gradient force in a hexagonal boron nitride slot waveguide. Opt. Lett..

[113] Yang Y., Finch M.F., Xiong D., Lail B.A. (2018). Hybrid long-range hyperbolic phonon polariton waveguide using hexagonal boron nitride for mid-infrared subwavelength confinement. Opt. Express.

[114] Miao S., Premkumar N., Yang Y., Xiong D., Lail B.A. (2019). Hybrid slot-waveguide fed antenna using hexagonal boron nitride D’yakonov polaritons. Opt. Express.

[115] Wu M., Yang Y., Fei H., Lin H., Han Y., Zhao X., Chen Z. (2022). Unidirectional transmission of visible region topological edge states in hexagonal boron nitride valley photonic crystals. Opt. Express.

[116] Qu T., Liu F., Lin Y., Cui K., Feng X., Zhang W., Huang Y. (2020). Cherenkov radiation generated in hexagonal boron nitride using extremely low-energy electrons. Nanophotonics.

[117] Ren W., Ouyang Y., Jiang P., Yu C., He J., Chen J. (2021). The Impact of Interlayer Rotation on Thermal Transport Across Graphene/Hexagonal Boron Nitride van der Waals Heterostructure. Nano Lett..

[118] Li P., Dolado I., Alfaro-Mozaz F.J., Casanova F., Hueso L.E., Liu S., Edgar J.H., Nikitin A.Y., Vélez S., Hillenbrand R. (2018). Infrared hyperbolic metasurface based on nanostructured van der Waals materials. Science.

[119] Feres F.H., Mayer R.A., Barcelos I.D., Freitas R.O., Maia F.C.B. (2020). Acceleration of Subwavelength Polaritons by Engineering Dielectric-Metallic Substrates. ACS Photonics.

[120] Li P., Hu G., Dolado I., Tymchenko M., Qiu C.W., Alfaro-Mozaz F.J., Casanova F., Hueso L.E., Liu S., Edgar J.H. (2020). Collective near-field coupling and nonlocal phenomena in infrared-phononic metasurfaces for nano-light canalization. Nat. Commun..

[121] Bylinkin A., Schnell M., Autore M., Calavalle F., Li P., Taboada-Gutièrrez J., Liu S., Edgar J.H., Casanova F., Hueso L.E. (2021). Real-space observation of vibrational strong coupling between propagating phonon polaritons and organic molecules. Nat. Photonics.

[122] Jia Y., Zhao H., Guo Q., Wang X., Wang H., Xia F. (2015). Tunable Plasmon–Phonon Polaritons in Layered Graphene–Hexagonal Boron Nitride Heterostructures. ACS Photonics.

[123] Wang L., Liu J., Ren B., Song J., Jiang Y. (2021). Tuning of mid-infrared absorption through phonon-plasmon-polariton hybridization in a graphene/hBN/graphene nanodisk array. Opt. Express.

[124] Guddala S., Komissarenko F., Kiriushechkina S., Vakulenko A., Li M., Menon V.M., Alù A., Khanikaev A.B. (2021). Topological phonon-polariton funneling in midinfrared metasurfaces. Science.

[125] Li Y., Xie X., Zeng H., Li B., Zhang Z., Wang S., Liu J., Shen D. (2022). Giant moiré trapping of excitons in twisted hBN. Opt. Express.

[126] Yasuda K., Wang X., Watanabe K., Taniguchi T., Jarillo-Herrero P. (2021). Stacking-engineered ferroelectricity in bilayer boron nitride. Science.

[127] Su C., Zhang F., Kahn S., Shevitski B., Jiang J., Dai C., Ungar A., Park J.H., Watanabe K., Taniguchi T. (2022). Tuning colour centres at a twisted hexagonal boron nitride interface. Nat. Mater..

[128] Shimatani M., Yamada N., Fukushima S., Okuda S., Ogawa S., Ikuta T., Maehashi K. (2019). High-responsivity turbostratic stacked graphene photodetectors using enhanced photogating. Appl. Phys. Express.

[129] Shimatani M., Ikuta T., Sakamoto Y., Fukushima S., Ogawa S., Maehashi K. (2022). Turbostratic stacked graphene-based high-responsivity mid-wavelength infrared detector using an enhanced photogating effect. Opt. Mater. Express.

[130] Akinwande D., Huyghebaert C., Wang C.H., Serna M.I., Goossens S., Li L.J., Wong H.P., Koppens F.H.L. (2019). Graphene and two-dimensional materials for silicon technology. Nature.

[131] Wang S., Liu X., Xu M., Liu L., Yang D., Zhou P. (2022). Two-dimensional devices and integration towards the silicon lines. Nat. Mater..

[132] Knobloch T., Illarionov Y.Y., Ducry F., Schleich C., Wachter S., Watanabe K., Taniguchi T., Mueller T., Waltl M., Lanza M. (2021). The performance limits of hexagonal boron nitride as an insulator for scaled CMOS devices based on two-dimensional materials. Nat. Electron..

[133] Lee J.S., Choi S.H., Yun S.J., Kim Y.I., Boandoh S., Park J.H., Shin B.G., Ko H., Lee S.H., Kim Y.M. (2018). Wafer-scale single-crystal hexagonal boron nitride film via self-collimated grain formation. Science.

[134] Uchida Y., Nakandakari S., Kawahara K., Yamasaki S., Mitsuhara M., Ago H. (2018). Controlled Growth of Large-Area Uniform Multilayer Hexagonal Boron Nitride as an Effective 2D Substrate. ACS Nano.

[135] Uchida Y., Kawahara K., Fukamachi S., Ago H. (2020). Chemical Vapor Deposition Growth of Uniform Multilayer Hexagonal Boron Nitride Driven by Structural Transformation of a Metal Thin Film. ACS Appl. Electron. Mater..

[136] Wang S., Crowther J., Kageshima H., Hibino H., Taniyasu Y. (2021). Epitaxial Intercalation Growth of Scalable Hexagonal Boron Nitride/Graphene Bilayer Moire Materials with Highly Convergent Interlayer Angles. ACS Nano.

[137] Wang P., Lee W., Corbett J.P., Koll W.H., Vu N.M., Laleyan D.A., Wen Q., Wu Y., Pandey A., Gim J. (2022). Scalable Synthesis of Monolayer Hexagonal Boron Nitride on Graphene with Giant Bandgap Renormalization. Adv. Mater..

[138] Seo Y., Masubuchi S., Watanabe E., Onodera M., Moriya R., Watanabe K., Taniguchi T., Machida T. (2020). Selective etching of hexagonal boron nitride by high-pressure CF4 plasma for individual one-dimensional ohmic contacts to graphene layers. Appl. Phys. Lett..

